# RETRACTION: Transcriptome Analysis Reveals Potential Mechanisms of the Effects of Dietary *Enteromorpha* Polysaccharides on Bursa of Fabricius in Broilers

**DOI:** 10.1002/vms3.1557

**Published:** 2024-07-17

S.‐J. Qiu, R. Zhang, Y. Guo, Y. Zhao, Z.‐H. Zhao, W.‐C. Liu, “Transcriptome Analysis Reveals Potential Mechanisms of the Effects of Dietary *Enteromorpha* Polysaccharides on Bursa of Fabricius in Broilers,” *Veterinary Medicine and Science* 7, no. 5 (2021): 1881–1889, https://doi.org/10.1002/vms3.573.

The above article, published online on 15 July 2021 in Wiley Online Library (wileyonlinelibrary.com) has been retracted by agreement between the journal's Editor‐in‐Chief, Gayle Hallowell, and John Wiley & Sons Ltd. An investigation by the publisher found this article to have experienced compromised peer review. Based on the investigation's findings, the Editor‐in‐Chief no longer has confidence in the reported results and conclusions drawn. Therefore, a decision has been made to retract this article. The authors have been informed of the decision to retract.

